# Immune Checkpoint Inhibitors for Unresectable Hepatocellular Carcinoma

**DOI:** 10.3390/vaccines8040616

**Published:** 2020-10-19

**Authors:** Mohamed A. Abd El Aziz, Antonio Facciorusso, Tarek Nayfeh, Samer Saadi, Mohamed Elnaggar, Christian Cotsoglou, Rodolfo Sacco

**Affiliations:** 1 Department of Surgery, Mayo Clinic, Rochester, MN 55905, USA; abdelmaksoud.mohamed@mayo.edu; 2 Gastroenterology Unit, Department of Medical Sciences, Ospedali Riuniti di Foggia, 71122 Foggia, Italy; antonio.facciorusso@virgilio.it; 3 Robert D. and Patricia E. Kern Center for the Science of Health Care Delivery, Mayo Clinic, Rochester, MN 55905, USA; Nayfeh.tarek@mayo.edu (T.N.); MohirSaadi.Samer@mayo.edu (S.S.); 4 Department of Internal Medicine, Reno School of Medicine, University of Nevada, Las Vegas, NV 1155, USA; melnaggar@med.unr.edu; 5 General Surgery Department, ASST-Vimercate, 20871 Vimercate, Italy; christian.cotsoglou@asst-vimercate.it; 6 Gastroenterology Unit, Department of Medical Sciences, Ospedali Riuniti di Foggia, Viale Pinto, 1, 71100 Foggia, Italy

**Keywords:** HCC, CPI, immunotherapy, survival, progression

## Abstract

Despite the advances in screening protocols and treatment options, hepatocellular carcinoma (HCC) is still considered to be the most lethal malignancy in patients with liver cirrhosis. Moreover, the survival outcomes after failure of first-line therapy for unresectable HCC is still poor with limited therapeutic options. One of these options is immune checkpoint inhibitors. The aim of this study is to comprehensively review the efficacy and safety of immune checkpoint inhibitors for patients with HCC.

## 1. Introduction

Hepatocellular carcinoma (HCC) is still the most common and most lethal malignancy in patients with liver cirrhosis, despite the advances in screening programs, chemoprophylaxis for high-risk patients and treatment options [1,2]. With the rapid increase in prevalence of metabolic disorders, nonalcoholic fatty liver disease became one of the leading risk factors of HCC after hepatitis B and C [3,4]. Overall, HCC is considered an inflammatory prototypic cancer. The high mortality rate from HCC is related to late diagnosis and the concomitant liver dysfunction. In that case, usually, curative resection or liver transplantation is not feasible [5].

Despite the recent advances in systemic therapy for unresectable HCC, patients who progress on first-line multikinase inhibitors, namely sorafenib [which targets vascular endothelial growth factor receptor (VEGFR), platelet-derived growth factor receptor (PDGFR-β) and rapidly accelerated fibrosarcoma (RAF) kinases] [6,7,8] and lenvatinib (which targets VEGFR1, VEGFR2 and VEGFR3, PDGFR alpha, fibroblast growth factor receptor (FGFR) and KIT and RET tyrosine kinases) [9,10,11,12,13], have limited options [5,14]. Moreover, these systemic therapies are usually associated with significant resistance and side-effects. Furthermore, some clinical trials designed to expand on the already existing options for patients with HCC showed disappointing results [15]. However, recently four additional targeted therapies got approval for treatment of HCC based on phase III randomized controlled trials. Those therapies include lenvatinib as first-line therapy [9] and regorafenib [14,16], cabozantinib [17] and ramucirumab [18] as rescue therapies after failure of sorafenib.

The tumor microenvironment of the HCC is infiltrated with different types of immune cells, mainly T-cells (CD8+, CD4+, Treg), natural killer cells and myeloid cells (myeloid-derived suppressor cells and tumor-associated macrophages). Due to the chronic inflammation and cirrhosis present in most HCC patients, the tumor ecosystem gets complicated affecting the behavior of the tumor and response to treatment. These changes are due to complex interactions between immune cells and tumor cells in the tumor microenvironment conveyed through cytokines and signaling pathways leading to exhaustion of pro-inflammatory immune cells and the dominance of the regulatory leukocytes hindering the anti-tumor response. A study by Yu et al. [19] concluded that improved overall survival was associated with high immune infiltration. The study further identified different immune clusters based on their prognostic value showing that better outcomes were associated with clusters with high levels of T-cells (mainly CD8+) and low levels of macrophages. A subset of tumor-associated macrophages (M1) was shown to be associated with improved outcomes. Poor prognosis is associated with the accumulation of myeloid-derived suppressor cells, tumor-associated macrophages, CD4+/CD25+/FOXP3+ immune-suppressive T-cells(T-reg), exhausted Th1 CD4+, CD8+ T-cells, dysfunctional NK cells and the expansion of Th2 CD4+ T-cells. Immune checkpoint molecules including programmed cell death (PD-1), CD274, cytotoxic T lymphocyte antigen -4 (CTLA-4), lymphocyte activated gene -3 (LAG-3) and IFNG were identified in clusters that had high levels of CD8+ T-cells. However, these clusters were associated with poor prognosis which leads to the assumption that these molecules are implicated in the HCC immune-exhaustion [20]. Therefore, it is assumed that the administration of immune checkpoint inhibitors would be beneficial for these HCC patients. In the United States, accelerated approval has been granted by the Food and Drug Administration (FDA) to two anti-programmed cell death monoclonal antibodies (nivolumab and pembrolizumab) and a combination of nivolumab plus ipilimumab, a monoclonal antibody against CTLA-4, for patients who progressed on sorafenib based on the results of several phase III trials [21,22,23]. However, data from phase III trial did not show superior efficacy of nivolumab as first-line therapy over sorafenib [24]. Moreover, the results of KEYNOTE 240 which assessed pembrolizumab as second-line therapy compared to placebo did not meet its predetermined level of statistical significance [25]. Therefore, we aimed to review the current evidence in the literature regarding the use of immune checkpoint inhibitors for the treatment of HCC.

## 2. Immune Checkpoint Inhibitors as a First-Line Therapy

### 2.1. PD-1/PD-L1 Inhibition

**Figure 1:**
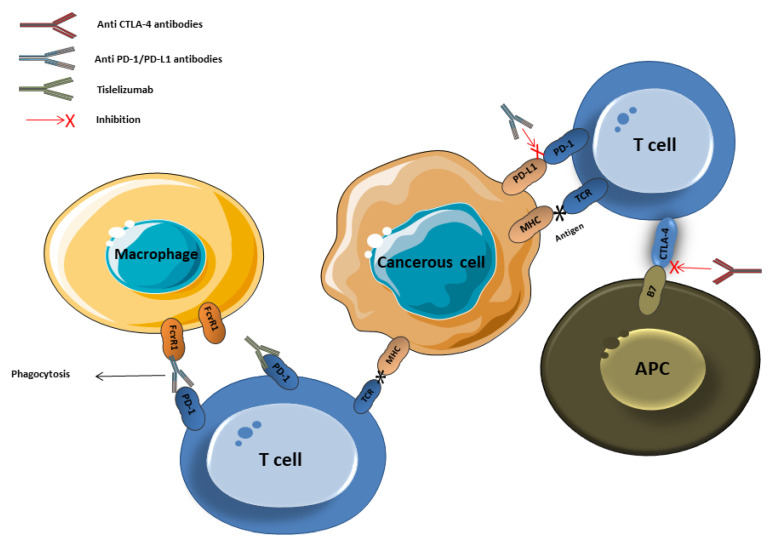
Immune checkpoints inhibitors’ mechanism of action. PD: programmed cell death, CTLA-4: cytotoxic T-lymphocyte associated protein 4, APC: antigen-presenting cell, MHC: major histocompatibility complex, TCR: T cell.

Avoiding immune destruction is one of the hallmarks of cancer. The PD-1/PD-L1 pathway plays a pivotal role in this escape mechanism [26]. Studies have shown that PD-L1 is overexpressed in tumor cells in different types of cancers including HCC, which leads to an increase in binding between PD-L1 and PD-1 on T cells within the tumor microenvironment resulting in immune anergy and apoptosis [27,28]. As a result, with overexpression of PD-L1, the tumor continues to grow unchecked which leads to worse prognosis in patients with HCC [28,29]. Interfering with this binding can result in enhancing immune reaction toward the cancer cells. (Figure 1) Therefore, the introduction of monoclonal antibody in the landscape of treatment of HCC has gained accelerated approval for patients who previously progressed on sorafenib based on the results CheckMate 040 trail [21]. However, for first-line therapy, the CheckMate 459 trial compared nivolumab to sorafenib in patients with Child-Pugh A (non-severe liver cirrhosis). Although the objective response rate was higher in the nivolumab group than the sorafenib group, the overall survival and progression-free survival were not significantly different between both groups [24] (Table 1).

One of the well-known mechanisms of resistance to anti-PD-1 therapy is FcɤR1 mediated macrophage antibody-dependent phagocytosis [30] (Figure 1). Therefore, another monoclonal antibody has been developed to evade the FcɤR1 mediated resistance that is, tislelizumab [31]. Clinical data from the RATIONAL 301 trial, which is comparing tislelizumab against sorafenib, supporting this mechanism are still pending [31].

### 2.2. Dual Immune Checkpoint Blockade

CTLA-4 is expressed on T regulatory cells regulating the early immune response after the primary stimulation by antigens mainly in lymphoid organs whereas PD-1 is expressed mainly on activated T cells in the tumor microenvironment regulating late immune response. Moreover, inhibition of the CTLA-4/B7 signal in lymph nodes increases activated CD8+ cells which will subsequently infiltrate the tumor and be part of the microenvironment [32,33]. Based on this, several studies have shown promising results with dual immunotherapy [34,35]. The success achieved in these trials especially in for patients with melanoma [36] has inspired the application of dual immune blockage for other types of cancers including HCC. Therefore, after the success achieved by the phase I/II trial investigating the efficacy and safety of dual immune therapy for patients progressed on sorafenib [37], a comparative randomized controlled trial, HIMALAYA study, was designed to compare Duravalumab versus the combination of Duravalumab plus Tremelimumab versus sorafenib. Its results are still bending (Table 1).

### 2.3. Combination with Biological Therapy

**Figure 2:**
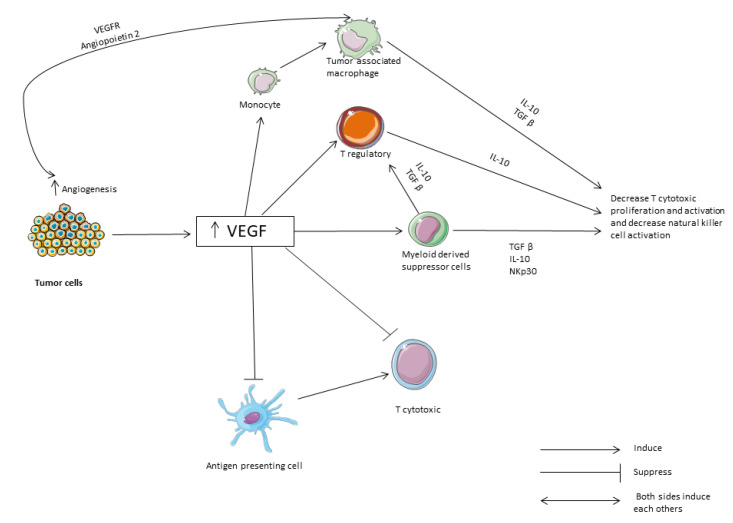
The role of VEGF and cytokines in immune suppression. VEGF: vascular endothelial growth factor. TGF: transformation growth factor.

Vascular endothelial growth factor (VEGF) has been linked with the development and progression of HCC [38,39]. Moreover, it has a role in immune suppression as it has been found that it creates an immunosuppressive microenvironment through the recruitment of several inhibitory cells such as T regulatory cells, tumor-associated macrophages and myeloid-derived suppressor cells. Those cells release cytokines such as IL-10 and TGF-β that inhibit natural killer cell and T cell activation and impedes dendritic cell maturation as shown in Figure 2 [33,40,41].

The landmark IMbrave 150 trial comparing atezolizumab (PD-L1 monoclonal antibody) plus bevacizumab (a monoclonal antibody against vascular endothelial growth factor) versus sorafenib found a better objective response rate and survival for patients treated with the combination therapy [42]. Moreover, the combination of atezolizumab plus bevacizumab showed a better progression-free survival when compared to atezolizumab alone [43]. As the main concern for patients with liver cirrhosis treated with bevacizumab is upper gastrointestinal bleeding, it occurred in 7% of the patients who received the combination therapy which is comparable to earlier reports evaluating bevacizumab alone in patients with HCC [42,44,45]. However, proteinuria and hypertension, as main side effects of bevacizumab, still among the top side effects of combination therapy. However, further evaluation of combination therapy versus sorafenib or lenvatinib as first-line therapy for HCC is still under investigation. For example, the combination of nivolumab plus ipilimumab versus sorafenib/lenvatinib as first-line therapy for HCC is still under investigation by the CheckMate 9DW trial (NCT04039607), the combination of cabozantinib plus atezolizumab versus sorafenib is under investigation by COSMIC 312 trial (NCT03755791) and the combination between pembrolizumab plus lenvatinib versus lenvatinib alone is under investigation by the LEAP 002 trial (NCT03713593). Nevertheless, the success achieved by the landmark IMbrave 150 trial and G030140 trial has a great implication for the practice regarding the upfront therapy for patients with unresectable HCC. Nevertheless, these trials included only patients with early liver disease and the efficacy and safety of the combination therapy in patients with advanced liver disease is still unelucidated. Furthermore, no data available about subsequent therapy after the failure of immune checkpoint. More details are provided in Table 1.

## 3. Immune Checkpoint Inhibitors as Second-Line Therapy

### 3.1. CTLA-4 Inhibition

Sangro et al. recruited 21 patients with hepatitis C virus who had progressed on previous lines of treatment for HCC. The treatment was tremelimumab at a dose of 15 mg/kg IV every 90 days. The drug showed a safe profile with a partial response rate of 17.6% [46]. Interestingly, the viral load for HCV decreased. Denoting the antiviral effect with the enhanced immunity. Moreover, the addition of ablation therapy to the anti-CTLA-4 showed a higher response rate with a similar safety profile [47] (Table 2).

### 3.2. PD-1/PD-L1 Inhibition

The progression-free survival, overall survival and response rates were found to be better for patients treated with anti-PD-1/PD-L1 compared to placebo [25]. However, the data from the retrospective analysis did not show differences between anti-PD-1/PD-L1 when compared to regorafenib [48,49]. Interestingly, a combination of anti-PD-1/PD-L1 with radiation therapy showed better progression-free survival and overall survival when compared to anti-PD-1/PDL-1 alone [50] (Table 2).

### 3.3. Dual Immune Checkpoint Blockade

Initial results of a single-arm study examining a combination between durvalumab and tremelimumab in patients with or without hepatitis infection. Out of 40 patients treated, 6 (15%) had an objective response rate with an acceptable safety profile [37] (Table 2).

### 3.4. Combination with Biological Therapy

Two studies evaluated the combination of biologic therapy with immunotherapy. The first evaluated ramucirumab plus duravalumab revealing an objective response rate of 11% and progression-free survival of 4.4 months [51]. The other one evaluated camrelizumab plus apatinib revealing an objective response rate of 44.4% and progression-free survival of 5.8 months [52] (Table 2).

## 4. Predictors of Response Using PD-L1 Expression

Immunohistochemical detection of PD-L1 has been studied in clinical trials as a predictor of response. It has been found that the expression of PD-L1 is associated with better overall response and survival outcomes [21,23]. A high tumor mutation burden (TMB), the number of somatic non-synchronous mutations in the genome of cancer cells, is a known predictive factor for response in different solid tumors. However, HCC has a low TMB compared to other solid malignancies which limited the predictive ability of this marker for HCC [53,54,55].

## 5. Immune Checkpoint Inhibitors for Subgroups of Patients

### 5.1. Use of Immune Checkpoint Inhibitors in Patients Autoimmune Diseases

One of the main concerns while treating patients with immune checkpoint inhibitors is immune-related adverse events which can be irreversible and even fatal [56,57,58]. Therefore, patients with a pre-existing auto-immune disease usually excluded from clinical trials [42], and, as a consequence, data about safety profiles in these populations is not available. However, liver cirrhosis can develop due to autoimmune diseases such as primary sclerosing cholangitis, autoimmune hepatitis, primary biliary cholangitis and so forth [59,60]. And, patients with HCC may suffer from another non-hepatobiliary autoimmune disease. Thus, understanding the underlying pathophysiological mechanisms and its interaction with the immune checkpoints’ pathways is crucial in order to provide these patients with the therapeutic advantages without devastating side effects.

Several retrospective studies and case reports evaluated the safety profile of immune checkpoint inhibitors for patients with cancer and concomitant autoimmune disease [61,62,63,64,65,66,67,68]. Abdel-Wahab et al. conducted a systematic review evaluating the safety of immune checkpoint in patients with preexisting autoimmune disease and they found that; although some events may be severe and even fatal, most immune flares and immune-related side effects are managed without permanent drug discontinuation. However, for patients with neurological diseases such as myasthenia graves and multiple sclerosis, almost all patients developed exacerbation or immune-related side effects. Therefore, careful evaluation should be considered before prescribing immune checkpoints inhibitors for patients with neurological autoimmune diseases [61]. In a more recent large scale study, patients with a preexisting autoimmune disease treated with immune checkpoints had a higher risk of immune-related side effects than the control group. Furthermore, active disease and female gender were found to be independent predictors for the development of immune-related side-effects [62].

In summary, the immune-related side effects seem to be higher in patients with pre-existing autoimmune disease. Active disease and female gender are independent risk factors for immune-related side effects. Although immune-related side effects in patients treated with immune checkpoint inhibitors with a pre-existing autoimmune disease can be fatal, most cases are managed successfully without permanent discontinuation of the immune checkpoints. Nevertheless, these observations are derived from case reports and small retrospective studies and a well-designed large scale trial still represents an unmet need. Moreover, data about patients with HCC carcinoma specifically and hepatobiliary autoimmune diseases is still sparse.

### 5.2. Use of Immune Checkpoint Inhibitors in Patients with Inflammatory Bowel Disease

**Figure 3:**
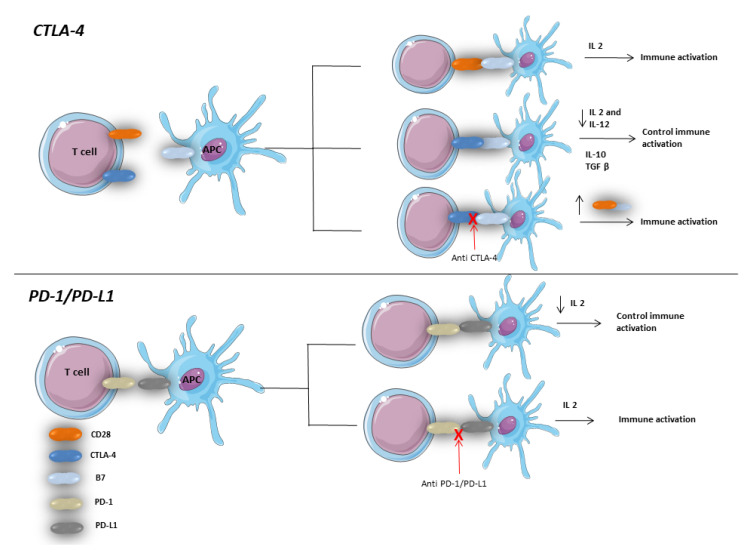
Role of CTLA-4 and PD-1/PD-L1 pathways in immune response regulation in gastrointestinal tract. APC: antigen-presenting cells.Both CD28 and CTLA-4 compete with each other for a binding site (B7) on the surface of APC. Binding of CD28 to B7 is associated with induction of immune response through upregulation of production of IL2. On the other side, CTLA-4 B7 binding regulates the late immune response by decreasing IL2 [71]. Therefore, inhibition of CTLA-4 by antiCTLA-4 antibodies was found to be associated with an exaggerated immune response which might lead to colitis [85,86]. PD-1/PD-L1 binding leads to immune response regulation through PI3K and AKT pathways. Therefore, inhibition of this binding might lead to immune response dysregulation which might lead to colitis and autoimmune exacerbation [87].

Patients suffering from inflammatory bowel disease (IBD) usually suffer from other hepatobiliary diseases such as drug-induced liver injury (about 30% of patients with IBD), primary sclerosing cholangitis (1.4% to 7.5% of patients with IBD), autoimmune hepatitis, primary biliary cirrhosis and nonalcoholic steatohepatitis [69]. These factors, along with the other traditional risk factors, can lead to HCC either directly or indirectly through liver cirrhosis [59]. Therefore, some patients who will suffer from HCC will have a concomitant IBD in which, as discussed before, immune checkpoint inhibitors with or without biologic therapy may be an option. However, the safety of immune checkpoint in this particular population is an ongoing and unanswered question. As known, the CTLA-4 and PD-1/PD-L1 signaling are crucial for gut homeostasis [70,71]. Interestingly, defects in the CTLA-4 gene or overexpression of PD-1/PD-L1 on intestinal epithelium were found to be higher in patients with IBD [72,73,74]. Figure 3 Therefore, in murine models, it was not surprising that the blockade of these pathways led to CD8 autoimmune enteritis [75]. And, it is not uncommon for a patient treated with immune checkpoint inhibitors to suffer from diarrhea [76]. Thus, IBD exacerbation during treatment with immune checkpoint inhibitors is a theoretical risk. Indeed, evidence about this question started to emanate from high volume centers. For example, in a recently published case series from Mayo Clinic, thirteen patients with a pre-diagnosed IBD were treated with immune checkpoint inhibitors and of them flare occurred in 4 patients (31%) [77]. This observation was also noted in a previous cohort in which 36% of patients with IBD treated with immune checkpoint inhibitors permanently discontinued them for IBD flare [68]. From a larger sample size study, data from a multicenter retrospective analysis included 102 patients with IBD treated with immune checkpoint inhibitors. Overall gastrointestinal side effects occurred in 42 patients (41%) after a median 62 days compared to 11% without IBD. Moreover, about 21% of patients with IBD treated with immune checkpoint inhibitors suffered from grade 3–4 diarrhea and 4 patients (3.9%) had intestinal perforation two of them had surgery [78]. Of note, the rate of intestinal perforation in patients receiving immune checkpoint inhibitors without concomitant IBD has been reported to be about 2.2% [79]. Importantly, most (~90%) of the included patients, in the aforementioned study evaluating the safety of immune checkpoints in patients with IBD, received a monotherapy of immune checkpoint inhibitors, only 10 patients (~10%) received a combination of two immune checkpoint inhibitors and none of the included patients received biologic therapy [78].

The combination of biologic therapy, especially bevacizumab, with immune checkpoint inhibitors in patients with IBD is of particular importance. Indeed, in patients not suffering from IBD, the intestinal perforation rate after using bevacizumab is about (1.5 to 2.5%) and severe bleeding is about 3% [80,81]. Importantly, the mortality rate for patients who develop intestinal perforation due to bevacizumab is high (up to 16%) [80,81]. Thus, even in non-gastrointestinal malignancies, the treatment with bevacizumab was found to be independently associated with a high risk of gastrointestinal perforation [82,83]. Indeed, the evidence about the safety of bevacizumab in patients with IBD is still lacking. Importantly, in patients with HCC, liver cirrhosis is common and gastrointestinal bleeding especially esophageal varices is a major concern, especially when selecting bevacizumab for treatment [84].

Overall, the use of atezolizumab with bevacizumab in patients with IBD carries a risk for intestinal perforation, gastrointestinal bleeding and the safety profile is still lacking in the literature.

## 6. Novel Immunotherapies

With the recent advances in the immunotherapeutic mechanisms, novel immunotherapies have gained popularity. Different therapeutic targets have been evaluated such as lymphocyte activation gene 3 (LAG-3). LAG-3 is first described by Triebel et al. and thereafter it was found to be overexpressed on the activated T cytotoxic and T regulatory cells with a negative impact on T helper cells. Therefore, during tumorigenesis, cancer cells use this pathway to escape from the immune system. Therefore, immunoglobin against LAG-3 has been investigated in different clinical trials [88]. Several other novel targets including T cell immunoglobulin and ITIM domain (TIGIT), T cell immunoglobulin and mucin domain-containing -3 (TIM-3) and B and T lymphocyte attenuator (BTLA) have been evaluated in clinical trials [88]. The current ongoing phase II trial [NCT03680508] is designed to evaluate the efficacy of TIM-3 in combination with PD-1 antibody for patients with HCC with no results published yet.

One other therapeutic target is the killer immunoglobulin-like receptor (KIR) which has an inhibitory effect on the NK cells. Therefore, Lirilumab, a KIR antibody, is under investigation in combination with immune checkpoint inhibitors in clinical trials [89].

Overall, the novel immunotherapies’ investigation in HCC is still restricted to being a part of evaluation of their role in solid tumors in general. Therefore, a better understanding of these pathways and their contribution to the HCC microenvironment is needed.

## 7. Conclusions

Hepatocellular carcinoma treatment represents a real challenge in patients with cirrhosis and several pharmacological [72,73,74,75] and loco-regional [76,77,78,79,80,81] therapies have been tested with mixed results. A combination of immune checkpoint inhibitors with biologic therapy seems to be promising for a new therapeutic standard of care for patients with unresectable HCC. However, for the subset of patients such as patients with preexisting autoimmune disease, inflammatory bowel disease or nonalcoholic steatohepatitis, the safety and efficacy are still not well established and further studies are needed to address all these open unanswered questions.

## Figures and Tables

**Table 1:** Immune checkpoint inhibitors as first-line therapy for unresectable hepatocellular carcinoma (HCC).

Study ID	NCT	Study Design, Key Inclusion	Sample Size	OS, Months (95% CI)	PFS Months (95% CI)	Response Rates	Side Effects
PD-1/PD-L1 antibodies							
CheckMate 459 ESMO October 2019	NCT02576509	RCT, CP: A	743 patients Nivolumab: 371 pts Sorafenib: 372 pts	Nivolumab vs. Sorafenib: OS: 16.4 (13.9–18.4) vs. 14.7 (11.9–17.2) 12 mo (%): 59.7 (54.4–64.6) vs. 55.1 (49.8–60.1) 24 mo (%): 36.8 (31.8–41.8) vs. 33.1 (28.3–38.0)	Nivolumab vs. Sorafenib: 3.7 (3.1–3.9) vs. 3.8 (3.7–4.5)	Nivolumab vs. Sorafenib: ORR: 57 (15%) vs. 26 (7%) Complete response: 14 (4%) vs. 5 (1%) Partial response: 43 (12%) vs. 21 (6%)	Nivolumab demonstrated a favorable safety profile consistent with previous reports.
RATIONALE 301	NCT03412773	RCT, BCLC stage C or B, CP: A	674 patients Tislelizumab vs. Sorafenib	Pending	Pending	Pending	Pending
Dual immune checkpoint blockade:							
HIMALAYA study	NCT03298451	RCT, BCLC stage C or B, CP: A	1310 pts, Durvalumab vs. (Durvalumab + Tremelimumab) vs. Sorafenib	Pending	Pending	Pending	Pending
Combination with biological therapy:							
IMbrave 150	NCT03434379	RCT, CP: A	501 patients Atezolizumab + Bevacizumab: 336 pts vs. Sorafenib: 165 pts	Atezolizumab + Bevacizumab vs. Sorafenib; Overall death: 28.6% vs. 39.4%; HR: 0.58 (95% CI 0.42–0.79) OS: NE vs. 13.2 (10.4—NE) OS at 6 Mo: 84.8% vs. 72.2%	Atezolizumab + Bevacizumab vs. Sorafenib; Overall progression: 58.6% vs. 66.1%; HR: 0.59 (95% CI 0.47–0.76) PFS: 6.8 (5.7–8.3) vs. 4.3 (4.0–5.6) PFS at 6 Mo: 57.5% vs. 37.2%	Atezolizumab + Bevacizumab vs. Sorafenib; % (95% CI) ORR per RECIST 1.1: 27.3 (22.5–32.5) vs. 11.9 (7.4–18) ORR per HCC specific mRECIST: 33.2 (28.1–38.6) vs. 13.3 (8.4–19.6)	Atezolizumab + Bevacizumab vs. Sorafenib; Grade 3–4 complications: 186 (56.5%) vs. 86 (55.1%)
G030140 group F	NCT02715531	RCT, CP: A	119 pts Atezolizumab + Bevacizumab: 60 pts vs. Atezolizumab: 59 pts	Atezolizumab + Bevacizumab vs. Atezolizumab Overall death: 27% vs. 31% OS: not reached in both groups	Atezolizumab + Bevacizumab vs. Atezolizumab Overall progression: HR: *per HCC mRECIST:* 57% vs. 66%, HR: 0.54 (80% CI 0.40–0.74) *per RECIST 1.1:* 58% vs. 66% HR: 0.55 (80% CI 0.40–0.74) PFS Mo: *per HCC mRECIST:* 5.6 mo (3.6–7.4) vs. 3.4 mo (1.9–5.2) *per RECIST:* 5.7 mo (3.5–9.3) vs. 2.0 mo (1.9–3.7)	Atezolizumab + Bevacizumab vs. Atezolizumab ORR per RECIST 1.1: 20% (95% CI 11–32) vs. 17% (95% CI 8–29) ORR per HCC mRECIST: 27% (95% CI 16–40) vs. 17% (95% CI 8–29)	Atezolizumab + Bevacizumab vs. Atezolizumab Grade 3–4: 12 (20%) vs. 3 (5%) The most common grade 3–4 SEs were: hypertension: 3 (5%) vs. none proteinuria: 2 (3%) vs. none
G030140 group A	NCT02715531	RCT, CP: A	104 pts Atezolizumab + Bevacizumab	57 (55%) still alive at data cut off OS not reached	Per RECIST 1.1: 66%; 7.3 months (95% CI 5.4–9.9) Per HCC mRECIST: 66%; 7.3 months (95% CI 5.4–9.9)	ORR per RECIST 1.1: n (%; 95% CI) 37 (36%; 26–46) ORR per HCC mRECIST: 41 (39%; 30–50)	Serious SEs: 25 (24%) The most common serious SEs were upper gastrointestinal hemorrhage, colitis, esophageal variceal hemorrhage and pneumonitis, each occurring in two (2%) patients.
COSMIC 312	NCT03755791	RCT, BCLC stage C or B, CP: A	740 pts Cabozantinib + Atezolizumab: 370 pts vs. Cabozantinib: 185 pts vs. Sorafenib: 185 pts	pending	pending	pending	Pending
LEAP 002	NCT03713593	RCT, BCLC stage C or B, CP: A	750 pts Pembrolizumab + Lenvatinib vs. Lenvatinib alone	pending	pending	pending	Pending
CheckMate 9DW	NCT04039607	RCT	1084 pts Nivolumab + Ipilimumab vs. Sorafenib/Lenvatinib	pending	pending	pending	Pending
KEYNOTE 524; AACR April 2019	NCT03006926	Single-arm, BCLC stage C or B, CP: A	104 pts will be recruited, however, the presented results are for 30 pts (6 pts in safety part and 24 pts in efficacy part) Pembrolizumab + Lenvatinib	pending	pending	ORR per mRECIST per investigator: 11 (36.7) per mRECIST per IIR: 15 (50.0%) Per RECIST IIR: 11 (36.7%)	Any-grade treatment-emergent adverse events (TEAEs) occurred in 28 pts (93%); the most common any-grade TEAEs were decreased appetite (63%) and hypertension (60%). 7 (23%) pts discontinued treatment due to TEAEs and no new safety signals were identified.
VEGF Liver 100	NCT03289533	Single-arm, BCLC stage C or B, CP: A	22 pts Avelumab + Axitinib	__	PFS: mo (95% CI) *Per RECIST:* 5.5 (1.9–7.3) *Per mRECIST:* 3.8 (1.9–7.3) 6 months PFS: % (95% CI) *Per RECIST:* 35.1% (15.3–55.8%) *Per mRECIST:* 30.9% (12.5–51.5%)	ORR *Per RECIST:* 13.6% (95% CI, 2.9–34.9%) *Per mRECIST:* 31.8% (95% CI, 13.9–54.9%)	The most common grade 3 treatment-related adverse events (TRAEs) (≥10% of patients) were hypertension (50.0%) and hand-foot syndrome (22.7%); no grade 4/5 TRAEs were reported.
Kelley 2017, arm five	NCT02519348	RCT	433 pts Durvalumab + Tremelimumab vs. Durvalumab vs. Tremelimumab vs. Durvalumab + Tremelimumab (regmine two) vs. Durvalumab + Bevacizumab	__	__	__	__

CP: Child-Pugh, RCT: Randomized Controlled Trial, RECIST: Response Evaluation Criteria in Solid Tumors, OS: Overall Survival, PFS: Progression-Free Survival, ORR: Objective Response Rate.

**Table 2:** Immune checkpoint inhibitors after failure or intolerability for first-line therapy for patients with unresectable HCC.

Study ID	NCT	Study Design, Key Inclusion	Sample Size	OS, Months (95% CI)	PFS Months (95% CI)	Response Rates	Side Effects
CTLA-4 antibodies							
Sangro 2013	NCT01008358	Single-arm, HCV patients, CP: A or B	21 pts Tremelimumab	__	__	ORR: 17.6% time to progression: 6.48 months (95% CI 3.95–9.14)	Grade 3–4 transaminase elevation: 45%
Duffy 2017	NCT01853618	Single-arm, CP: A or B	32 pts Tremelimumab plus ablation	OS: 12.3 months (95% CI 9.3 to 15.4 months). Six months OS: 85.7% (66.3–94.4%) One year OS: 50.8% (29.1–68.9%)	PFS: 7.4 months (4.7–19.4 months) Six months PFS: 57.1% (37.1–72.9%) One year PFS: 33.1% (16.2–51.2%)	Partial response: 26% (95% CI 9.1–51.2)	Grade 3–4 increase AST: 7 pts (19%)
PD-1/PD-L1 inhibition:							
KEYNOTE 240	NCT02702401	RCT, CP: A	413 pts Pembrolizumab: 278 pts vs. Placebo: 135 pts	OS: Pembrolizumab: 13.9 months (95% CI, 11.6 to 16.0 months) Placebo: 10.6 months (95% CI, 8.3 to 13.5 months) HR: 0.781; 95% CI, 0.611 to 0.998	PFS: Pembrolizumab: 3.0 months (95% CI, 2.8 to 4.1 months) Placebo: 2.8 months (95% CI, 1.6 to 3.0 months) HR: 0.718; 95% CI, 0.570 to 0.904 PFS at 12 months: Pembrolizumab: 19.4% (95% CI, 14.6% to 24.9%) Placebo: 6.7% (95% CI, 3.0% to 12.4%)	ORR: Pembrolizumab: 18.3% (95% CI 14–23.4) Placebo: 4.4% (95% CI 1.6–9.4) Estimated treatment difference: 13.8 (95% CI: 7.7 to 19.5)	Any grade 3–4: Pembrolizumab: 52% Placebo: 46.3% Grade 3–4 AST elevation: Pembrolizumab: 13.3% Placebo: 7.5%
Scheiner 2019	NA	Retrospective cohort	65 pts Nivolumab: 34 pts Pembrolizumab: 31 pts	OS: Nivolumab: 9.0 (95% CI, 5.5–12.5) months Pembrolizumab: 11.0 (95% CI, 7.4–14.5) months 1 year OS: Nivolumab: 38% Pembrolizumab: 44%	PFS Nivolumab: 4.3 (95% CI, 2.0–6.7) months Pembrolizumab: 5.6 (95% CI, 1.1–10.1) months	ORR: Nivolumab: 15% Pembrolizumab: 10%	High grade: 17% in both groups
Choi 2020	NA	Propensity score matching, CP: A	272 pts after matching Regorafenib: 136 pts vs. Nivolumab: 136 pts	weeks, median (95% CI) Regorafenib: 31.3 (24.6–42.0) Nivolumab: 37.1 (22.4–49.0)	time in weeks; median (95% CI) Regorafenib: 12.6 (10.6–15.7) Nivolumab: 7.1 (6.1–11.1)	ORR: Regorafenib: 3.7% Nivolumab: 14%	
Lee 2020	NA	Retrospective cohort	150 patients Regorafenib: 102 patients Nivolumab: 48 patients	OS: Regorafenib: 6.9 months (95% CI, 3.5–13.1) Nivolumab: 5.9 months (95% CI, 3.2–18.1) Death rates: Regorafenib: 37.3% Nivolumab: 56.3%	mTTP Regorafenib: 3.3 months; (95% CI, 2.0–5.3) Nivolumab: 4.0 months; (95% CI, 1.8–8.7) Progression: Regorafenib: 60.8% Nivolumab: 60.4%	ORR: Regorafenib: 5.9% Nivolumab: 16.7%	
Yu 2019	NA	Retrospective cohort	76 pts Nivolumab alone: 22 pts Nivolumab plus radiotherapy: 54 pts	Patients who had received previous/concurrent RT had a significantly longer progression-free survival (PFS; *p* = 0.008) and overall survival (OS; *p* = 0.007) than those who did not receive RT	__	No complete response PR: Nivolumab alone: 1 pt (4.5%) Nivolumab plus radio: 8 pts (14.8%)	Nivolumab-related toxicities were generally tolerable regardless of the history of RT.
Qin 2020	NCT02989922	RCT	Total 220 pts Camrelizumab every two weeks group: 111 pts Camrelizumab every three weeks group: 109 pts.	OS: Overall: 13.8 (11.5–16.6) Two months: 14.2 (11.5–NR) three months: 13.2 (9.4–17.0) OS rates: At 6 months, % (95% CI): Overall: 74.4% (68.0–79.7) Two weeks: 75.9% (66.6–82.9) Three weeks: 73.0% (63.6–80.4) At 9 months: Overall: 64.0% (57.2–70.1) Two weeks: 67.3% (57.5–75.3) Three weeks: 60.8% (50.8–69.3) At 12 months: Overall: 55.9% (48.9–62.2) Two weeks: 59.6% (49.6–68.2) Three weeks:52.2% (42.3–61.2)	PFS: Overall: 2.1 months (2.0–3.2) Two weeks: 2.3 months (1.9–3.2) Three weeks: 2.0 months (2.0–3.2) Disease progression rate: Overall: 73% Two weeks: 72% Three weeks: 74%	ORR: Number (%, 95% CI) Overall: 32 (14.7%; 10.3–20.2) Every two weeks: 13 (11.9%; 6.5–19.5) Every three weeks: 19 (17.6%; 10.9–26.1)	Grade 3: Overall: 11 (5.1%) Two weeks: 11 (10.1%) Three weeks: 6 (5.6%) Grade 4: Overall: 5 (2.3%) Two weeks: zero Three weeks: zero (I do not know how both two weeks and three weeks are zero but ht overall is 5) Grade five: Overall: 1 (0.5%), two and three weeks are zero.
CHECKMATE 040	Dose escalation	Phase I/2 trial	48 pts Nivolumab	__	__	__	Treatment-related grade 3–4: 25%
	Dose expansion		214 pts Nivolumab uninfected Sorafenib untreated/intolerant: 56 pts uninfected Sorafenib progressors: 57 pts HCV: 50 pts HBV: 51 pts	OS: not reached 6 months OS: Overall: 83% (78 to 88) uninfected untreated/intolerant: 89% (77 to 95) uninfected Sorafenib progressors: 75% (62 to 85) HCV: 85% (72 to 93) HBV: 84% (71 to 92)	PFS: Overall: 4.0 (2.9 to 5.4) uninfected untreated/intolerant: 5.4 (3.9 to 8.5) uninfected Sorafenib progressors: 4.0 (2.6 to 6.7) HCV: 4.0 (2.6 to 5.7) HBV: 4.0 (1.3 to 4.1)	ORR: Overall: 42 (20%; 15 to 26) uninfected untreated/intolerant: 13 (23%; 13 to 36) uninfected Sorafenib progressors: 12 (21%; 11 to 34) HCV: 10 (20%; 10 to 34) HBV: 7 (14%; 6 to 26)	Grade 3–4: (19%)
KEYNOTE 224	NCT02702414	Single-arm, CP: A	104 pts Pembrolizumab	OS: 12.9 months (95% CI 9.7–15.5) OS at 12 months: 54% (95% CI 44–63)	PFS: 4.9 months (95% CI 3.4–7.2) PFS at 12 months: 28% (95% CI 19–37)	ORR: 17% (95% CI 11–26)	Grade 3: 24%
He 2018	NCT02383212	Single-arm, CP: A	26 pts Cemiplimab	__	PFS: 3.7 months (95% CI: 2.3–9.1)	PR: 19.2% Stable disease: 53.8%	1 death due to hepatic failure related to treatment
	NCT04294498	Single-arm, HBV, CP: A	43 pts Durvalumab	__	__	__	__
Dual immune checkpoint blockade							
Kelley 2017	NCT02519348	RCT, here we present the results of initial phase one safety and efficacy analysis	40 pts Durvalumab/Tremelimumab combination	__	__	ORR: 15%	Most common grade ≥3 related AE was asymptomatic increased AST (10%)
Combination with biological therapy:							
Bang 2019	NCT02572687	Single-arm	28 pts Ramucirumab and Durvalumab	10.7 months (95% CI 5.1–18.4)	4.4 months (95% CI 1.6–5.7)	ORR: 3 (11%)	
Xu 2019	NCT02942329	Single-arm	18 pts Camrelizumab + Apatinib	OS: not reached	PFS: 5.8 months (2.6, NR) At 6 months: 45.4% (20.9%, 67.1%) At 9 months: 37.8% (15.0%, 60.7%)	ORR: 44.4%	

CP: Child-Pugh, RCT: Randomized Controlled Trial, RECIST: Response Evaluation Criteria in Solid Tumors, OS: Overall Survival, PFS: Progression-Free Survival, ORR: Objective Response Rate, TTP: Time to Progression, PR: Partial Response.

## References

[1] El-Serag H.B. (2011). Hepatocellular carcinoma. N. Engl. J. Med..

[2] Abd El Aziz M.A., Sacco R., Facciorusso A. (2020). Nucleos(t)ide analogues and Hepatitis B virus-related hepatocellular carcinoma: A literature review. Antivir. Chem. Chemother..

[3] Kim D., Li A.A., Perumpail B.J., Gadiparthi C., Kim W., Cholankeril G., Glenn J.S., Harrison S.A., Younossi Z.M., Ahmed A. (2019). Changing Trends in Etiology-Based and Ethnicity-Based Annual Mortality Rates of Cirrhosis and Hepatocellular Carcinoma in the United States. Hepatology.

[4] Facciorusso A., Abd El Aziz M.A., Singh S., Pusceddu S., Milione M., Giacomelli L., Sacco R. (2020). Statin Use Decreases the Incidence of Hepatocellular Carcinoma: An Updated Meta-Analysis. Cancers.

[5] Kudo M., Trevisani F., Abou-Alfa G.K., Rimassa L. (2016). Hepatocellular Carcinoma: Therapeutic Guidelines and Medical Treatment. Liver Cancer.

[6] Llovet J.M., Ricci S., Mazzaferro V., Hilgard P., Gane E., Blanc J.F., de Oliveira A.C., Santoro A., Raoul J.L., Forner A. (2008). Sorafenib in advanced hepatocellular carcinoma. N. Engl. J. Med..

[7] Wilhelm S.M., Adnane L., Newell P., Villanueva A., Llovet J.M., Lynch M. (2008). Preclinical overview of sorafenib, a multikinase inhibitor that targets both Raf and VEGF and PDGF receptor tyrosine kinase signaling. Mol. Cancer Ther..

[8] Keating G.M., Santoro A. (2009). Sorafenib. Drugs.

[9] Kudo M., Finn R.S., Qin S., Han K.H., Ikeda K., Piscaglia F., Baron A., Park J.W., Han G., Jassem J. (2018). Lenvatinib versus sorafenib in first-line treatment of patients with unresectable hepatocellular carcinoma: A randomised phase 3 non-inferiority trial. Lancet.

[10] Matsui J., Funahashi Y., Uenaka T., Watanabe T., Tsuruoka A., Asada M. (2008). Multi-kinase inhibitor E7080 suppresses lymph node and lung metastases of human mammary breast tumor MDA-MB-231 via inhibition of vascular endothelial growth factor-receptor (VEGF-R) 2 and VEGF-R3 kinase. Clin. Cancer Res..

[11] Okamoto K., Kodama K., Takase K., Sugi N.H., Yamamoto Y., Iwata M., Tsuruoka A. (2013). Antitumor activities of the targeted multi-tyrosine kinase inhibitor lenvatinib (E7080) against RET gene fusion-driven tumor models. Cancer Lett..

[12] Matsui J., Yamamoto Y., Funahashi Y., Tsuruoka A., Watanabe T., Wakabayashi T., Uenaka T., Asada M. (2008). E7080, a novel inhibitor that targets multiple kinases, has potent antitumor activities against stem cell factor producing human small cell lung cancer H146, based on angiogenesis inhibition. Int. J. Cancer.

[13] Yamamoto Y., Matsui J., Matsushima T., Obaishi H., Miyazaki K., Nakamura K., Tohyama O., Semba T., Yamaguchi A., Hoshi S.S. (2014). Lenvatinib, an angiogenesis inhibitor targeting VEGFR/FGFR, shows broad antitumor activity in human tumor xenograft models associated with microvessel density and pericyte coverage. Vasc. Cell.

[14] Facciorusso A., Abd El Aziz M.A., Sacco R. (2019). Efficacy of Regorafenib in Hepatocellular Carcinoma Patients: A Systematic Review and Meta-Analysis. Cancers.

[15] Pinter M., Peck-Radosavljevic M. (2018). Review article: Systemic treatment of hepatocellular carcinoma. Aliment. Pharmacol. Ther..

[16] Bruix J., Qin S., Merle P., Granito A., Huang Y.H., Bodoky G., Pracht M., Yokosuka O., Rosmorduc O., Breder V. (2017). Regorafenib for patients with hepatocellular carcinoma who progressed on sorafenib treatment (RESORCE): A randomised, double-blind, placebo-controlled, phase 3 trial. Lancet.

[17] Abou-Alfa G.K., Meyer T., Cheng A.-L., El-Khoueiry A.B., Rimassa L., Ryoo B.-Y., Cicin I., Merle P., Chen Y., Park J.-W. (2018). Cabozantinib in Patients with Advanced and Progressing Hepatocellular Carcinoma. N. Engl. J. Med..

[18] Zhu A.X., Kang Y.K., Yen C.J., Finn R.S., Galle P.R., Llovet J.M., Assenat E., Brandi G., Pracht M., Lim H.Y. (2019). Ramucirumab after sorafenib in patients with advanced hepatocellular carcinoma and increased α-fetoprotein concentrations (REACH-2): A randomised, double-blind, placebo-controlled, phase 3 trial. Lancet Oncol..

[19] Yu S., Wang Y., Hou J., Li W., Wang X., Xiang L., Tan D., Wang W., Jiang L., Claret F.X. (2020). Tumor-infiltrating immune cells in hepatocellular carcinoma: Tregs is correlated with poor overall survival. PLoS ONE.

[20] Pinato D.J., Guerra N., Fessas P., Murphy R., Mineo T., Mauri F.A., Mukherjee S.K., Thursz M., Wong C.N., Sharma R. (2020). Immune-based therapies for hepatocellular carcinoma. Oncogene.

[21] El-Khoueiry A.B., Sangro B., Yau T., Crocenzi T.S., Kudo M., Hsu C., Kim T.Y., Choo S.P., Trojan J., Welling T.H.R. (2017). Nivolumab in patients with advanced hepatocellular carcinoma (CheckMate 040): An open-label, non-comparative, phase 1/2 dose escalation and expansion trial. Lancet.

[22] Yau T., Kang Y.-K., Kim T.-Y., El-Khoueiry A.B., Santoro A., Sangro B., Melero I., Kudo M., Hou M.-M., Matilla A. (2019). Nivolumab (NIVO) + ipilimumab (IPI) combination therapy in patients (pts) with advanced hepatocellular carcinoma (aHCC): Results from CheckMate 040. J. Clin. Oncol..

[23] Zhu A.X., Finn R.S., Edeline J., Cattan S., Ogasawara S., Palmer D., Verslype C., Zagonel V., Fartoux L., Vogel A. (2018). Pembrolizumab in patients with advanced hepatocellular carcinoma previously treated with sorafenib (KEYNOTE-224): A non-randomised, open-label phase 2 trial. Lancet Oncol..

[24] Yau T., Park J.W., Finn R.S., Cheng A.L., Mathurin P., Edeline J., Kudo M., Han K.H., Harding J.J., Merle P. (2019). CheckMate 459: A randomized, multi-center phase III study of nivolumab (NIVO) vs sorafenib (SOR) as first-line (1L) treatment in patients (pts) with advanced hepatocellular carcinoma (aHCC). Ann. Oncol..

[25] Finn R.S., Ryoo B.Y., Merle P., Kudo M., Bouattour M., Lim H.Y., Breder V., Edeline J., Chao Y., Ogasawara S. (2020). Pembrolizumab As Second-Line Therapy in Patients With Advanced Hepatocellular Carcinoma in KEYNOTE-240: A Randomized, Double-Blind, Phase III Trial. J. Clin. Oncol. Off. J. Am. Soc. Clin. Oncol..

[26] Hanahan D., Weinberg R.A. (2011). Hallmarks of cancer: The next generation. Cell.

[27] Khan H., Gucalp R., Shapira I. (2015). Evolving Concepts: Immunity in Oncology from Targets to Treatments. J. Oncol..

[28] Shi F., Shi M., Zeng Z., Qi R.Z., Liu Z.W., Zhang J.Y., Yang Y.P., Tien P., Wang F.S. (2011). PD-1 and PD-L1 upregulation promotes CD8(+) T-cell apoptosis and postoperative recurrence in hepatocellular carcinoma patients. Int. J. Cancer.

[29] Gao Q., Wang X.Y., Qiu S.J., Yamato I., Sho M., Nakajima Y., Zhou J., Li B.Z., Shi Y.H., Xiao Y.S. (2009). Overexpression of PD-L1 significantly associates with tumor aggressiveness and postoperative recurrence in human hepatocellular carcinoma. Clin. Cancer Res..

[30] Zhang T., Song X., Xu L., Ma J., Zhang Y., Gong W., Zhang Y., Zhou X., Wang Z., Wang Y. (2018). The binding of an anti-PD-1 antibody to FcγRΙ has a profound impact on its biological functions. Cancer Immunol. Immunother. CII.

[31] Qin S., Finn R.S., Kudo M., Meyer T., Vogel A., Ducreux M., Macarulla T.M., Tomasello G., Boisserie F., Hou J. (2019). RATIONALE 301 study: Tislelizumab versus sorafenib as first-line treatment for unresectable hepatocellular carcinoma. Future Oncol..

[32] Iwai Y., Hamanishi J., Chamoto K., Honjo T. (2017). Cancer immunotherapies targeting the PD-1 signaling pathway. J. Biomed. Sci..

[33] Kudo M. (2019). Combination Cancer Immunotherapy with Molecular Targeted Agents/Anti-CTLA-4 Antibody for Hepatocellular Carcinoma. Liver Cancer.

[34] Wing K., Onishi Y., Prieto-Martin P., Yamaguchi T., Miyara M., Fehervari Z., Nomura T., Sakaguchi S. (2008). CTLA-4 control over Foxp3+ regulatory T cell function. Science.

[35] Kudo M. (2019). Immuno-Oncology Therapy for Hepatocellular Carcinoma: Current Status and Ongoing Trials. Liver Cancer.

[36] Postow M.A., Chesney J., Pavlick A.C., Robert C., Grossmann K., McDermott D., Linette G.P., Meyer N., Giguere J.K., Agarwala S.S. (2015). Nivolumab and ipilimumab versus ipilimumab in untreated melanoma. N. Engl. J. Med..

[37] Kelley R.K., Abou-Alfa G.K., Bendell J.C., Kim T.-Y., Borad M.J., Yong W.-P., Morse M., Kang Y.-K., Rebelatto M., Makowsky M. (2017). Phase I/II study of durvalumab and tremelimumab in patients with unresectable hepatocellular carcinoma (HCC): Phase I safety and efficacy analyses. J. Clin. Oncol..

[38] Morse M.A., Sun W., Kim R., He A.R., Abada P.B., Mynderse M., Finn R.S. (2019). The Role of Angiogenesis in Hepatocellular Carcinoma. Clin. Cancer Res. Off. J. Am. Assoc. Cancer Res..

[39] Zhu A.X., Duda D.G., Sahani D.V., Jain R.K. (2011). HCC and angiogenesis: Possible targets and future directions. Nat. Rev. Clin. Oncol..

[40] Voron T., Marcheteau E., Pernot S., Colussi O., Tartour E., Taieb J., Terme M. (2014). Control of the immune response by pro-angiogenic factors. Front. Oncol..

[41] Fukumura D., Kloepper J., Amoozgar Z., Duda D.G., Jain R.K. (2018). Enhancing cancer immunotherapy using antiangiogenics: Opportunities and challenges. Nat. Rev. Clin. Oncol..

[42] Finn R.S., Qin S., Ikeda M., Galle P.R., Ducreux M., Kim T.Y., Kudo M., Breder V., Merle P., Kaseb A.O. (2020). Atezolizumab plus Bevacizumab in Unresectable Hepatocellular Carcinoma. N. Engl. J. Med..

[43] Lee M.S., Ryoo B.Y., Hsu C.H., Numata K., Stein S., Verret W., Hack S.P., Spahn J., Liu B., Abdullah H. (2020). Atezolizumab with or without bevacizumab in unresectable hepatocellular carcinoma (GO30140): An open-label, multicentre, phase 1b study. Lancet Oncol..

[44] Pinter M., Ulbrich G., Sieghart W., Kölblinger C., Reiberger T., Li S., Ferlitsch A., Müller C., Lammer J., Peck-Radosavljevic M. (2015). Hepatocellular Carcinoma: A Phase II Randomized Controlled Double-Blind Trial of Transarterial Chemoembolization in Combination with Biweekly Intravenous Administration of Bevacizumab or a Placebo. Radiology.

[45] Siegel A.B., Cohen E.I., Ocean A., Lehrer D., Goldenberg A., Knox J.J., Chen H., Clark-Garvey S., Weinberg A., Mandeli J. (2008). Phase II trial evaluating the clinical and biologic effects of bevacizumab in unresectable hepatocellular carcinoma. J. Clin. Oncol. Off. J. Am. Soc. Clin. Oncol..

[46] Sangro B., Gomez-Martin C., de la Mata M., Iñarrairaegui M., Garralda E., Barrera P., Riezu-Boj J.I., Larrea E., Alfaro C., Sarobe P. (2013). A clinical trial of CTLA-4 blockade with tremelimumab in patients with hepatocellular carcinoma and chronic hepatitis C. J. Hepatol..

[47] Duffy A.G., Ulahannan S.V., Makorova-Rusher O., Rahma O., Wedemeyer H., Pratt D., Davis J.L., Hughes M.S., Heller T., ElGindi M. (2017). Tremelimumab in combination with ablation in patients with advanced hepatocellular carcinoma. J. Hepatol..

[48] Choi W.M., Choi J., Lee D., Shim J.H., Lim Y.S., Lee H.C., Chung Y.H., Lee Y.S., Park S.R., Ryu M.H. (2020). Regorafenib Versus Nivolumab After Sorafenib Failure: Real-World Data in Patients With Hepatocellular Carcinoma. Hepatol. Commun..

[49] Lee C.H., Lee Y.B., Kim M.A., Jang H., Oh H., Kim S.W., Cho E.J., Lee K.H., Lee J.H., Yu S.J. (2020). Effectiveness of nivolumab versus regorafenib in hepatocellular carcinoma patients who failed sorafenib treatment. Clin. Mol. Hepatol..

[50] Yu J.I., Lee S.J., Lee J., Lim H.Y., Paik S.W., Yoo G.S., Choi C., Park H.C. (2019). Clinical significance of radiotherapy before and/or during nivolumab treatment in hepatocellular carcinoma. Cancer Med..

[51] Bang Y.-J., Golan T., Lin C.-C., Dahan L., Fu S., Moreno V., Geva R., Reck M., Wasserstrom H.A., Mi G. (2019). Ramucirumab (Ram) and durvalumab (Durva) treatment of metastatic non-small cell lung cancer (NSCLC), gastric/gastroesophageal junction (G/GEJ) adenocarcinoma, and hepatocellular carcinoma (HCC) following progression on systemic treatment(s). J. Clin. Oncol..

[52] Xu J., Zhang Y., Jia R., Yue C., Chang L., Liu R., Zhang G., Zhao C., Zhang Y., Chen C. (2019). Anti-PD-1 Antibody SHR-1210 Combined with Apatinib for Advanced Hepatocellular Carcinoma, Gastric, or Esophagogastric Junction Cancer: An Open-label, Dose Escalation and Expansion Study. Clin. Cancer Res. Off. J. Am. Assoc. Cancer Res..

[53] Totoki Y., Tatsuno K., Covington K.R., Ueda H., Creighton C.J., Kato M., Tsuji S., Donehower L.A., Slagle B.L., Nakamura H. (2014). Trans-ancestry mutational landscape of hepatocellular carcinoma genomes. Nat. Genet..

[54] Yarchoan M., Hopkins A., Jaffee E.M. (2017). Tumor Mutational Burden and Response Rate to PD-1 Inhibition. N. Engl. J. Med..

[55] Zucman-Rossi J., Villanueva A., Nault J.C., Llovet J.M. (2015). Genetic Landscape and Biomarkers of Hepatocellular Carcinoma. Gastroenterology.

[56] Haanen J., Carbonnel F., Robert C., Kerr K.M., Peters S., Larkin J., Jordan K. (2017). Management of toxicities from immunotherapy: ESMO Clinical Practice Guidelines for diagnosis, treatment and follow-up. Ann. Oncol. Off. J. Eur. Soc. Med. Oncol..

[57] Konstantina T., Konstantinos R., Anastasios K., Anastasia M., Eleni L., Ioannis S., Sofia A., Dimitris M. (2019). Fatal adverse events in two thymoma patients treated with anti-PD-1 immune check point inhibitor and literature review. Lung Cancer.

[58] Krenn M., Grisold A., Wohlfarth P., Rath J., Cetin H., Koneczny I., Zimprich F. (2020). Pathomechanisms and Clinical Implications of Myasthenic Syndromes Exacerbated and Induced by Medical Treatments. Front. Mol. Neurosci..

[59] Herbst D.A., Reddy K.R. (2012). Risk factors for hepatocellular carcinoma. Clin. Liver Dis..

[60] Tansel A., Katz L.H., El-Serag H.B., Thrift A.P., Parepally M., Shakhatreh M.H., Kanwal F. (2017). Incidence and Determinants of Hepatocellular Carcinoma in Autoimmune Hepatitis: A Systematic Review and Meta-analysis. Clin. Gastroenterol. Hepatol. Off. Clin. Pract. J. Am. Gastroenterol. Assoc..

[61] Abdel-Wahab N., Shah M., Lopez-Olivo M.A., Suarez-Almazor M.E. (2018). Use of Immune Checkpoint Inhibitors in the Treatment of Patients With Cancer and Preexisting Autoimmune Disease: A Systematic Review. Ann. Intern. Med..

[62] Cortellini A., Buti S., Santini D., Perrone F., Giusti R., Tiseo M., Bersanelli M., Michiara M., Grassadonia A., Brocco D. (2019). Clinical Outcomes of Patients with Advanced Cancer and Pre-Existing Autoimmune Diseases Treated with Anti-Programmed Death-1 Immunotherapy: A Real-World Transverse Study. Oncologist.

[63] Danlos F.X., Voisin A.L., Dyevre V., Michot J.M., Routier E., Taillade L., Champiat S., Aspeslagh S., Haroche J., Albiges L. (2018). Safety and efficacy of anti-programmed death 1 antibodies in patients with cancer and pre-existing autoimmune or inflammatory disease. Eur. J. Cancer.

[64] Johnson D.B., Sullivan R.J., Ott P.A., Carlino M.S., Khushalani N.I., Ye F., Guminski A., Puzanov I., Lawrence D.P., Buchbinder E.I. (2016). Ipilimumab Therapy in Patients With Advanced Melanoma and Preexisting Autoimmune Disorders. JAMA Oncol..

[65] Kähler K.C., Eigentler T.K., Gesierich A., Heinzerling L., Loquai C., Meier F., Meiss F., Pföhler C., Schlaak M., Terheyden P. (2018). Ipilimumab in metastatic melanoma patients with pre-existing autoimmune disorders. Cancer Immunol. Immunother. CII.

[66] Leonardi G.C., Gainor J.F., Altan M., Kravets S., Dahlberg S.E., Gedmintas L., Azimi R., Rizvi H., Riess J.W., Hellmann M.D. (2018). Safety of Programmed Death-1 Pathway Inhibitors Among Patients With Non-Small-Cell Lung Cancer and Preexisting Autoimmune Disorders. J. Clin. Oncol. Off. J. Am. Soc. Clin. Oncol..

[67] Menzies A.M., Johnson D.B., Ramanujam S., Atkinson V.G., Wong A.N.M., Park J.J., McQuade J.L., Shoushtari A.N., Tsai K.K., Eroglu Z. (2017). Anti-PD-1 therapy in patients with advanced melanoma and preexisting autoimmune disorders or major toxicity with ipilimumab. Ann. Oncol. Off. J. Eur. Soc. Med. Oncol..

[68] Tison A., Quéré G., Misery L., Funck-Brentano E., Danlos F.X., Routier E., Robert C., Loriot Y., Lambotte O., Bonniaud B. (2019). Safety and Efficacy of Immune Checkpoint Inhibitors in Patients With Cancer and Preexisting Autoimmune Disease: A Nationwide, Multicenter Cohort Study. Arthritis Rheumatol..

[69] Rojas-Feria M., Castro M., Suárez E., Ampuero J., Romero-Gómez M. (2013). Hepatobiliary manifestations in inflammatory bowel disease: The gut, the drugs and the liver. World J. Gastroenterol..

[70] Dougan M. (2017). Checkpoint Blockade Toxicity and Immune Homeostasis in the Gastrointestinal Tract. Front. Immunol..

[71] Chambers C.A., Allison J.P. (1999). Costimulatory regulation of T cell function. Curr. Opin. Cell Biol..

[72] Ng S.C., Tsoi K.K., Kamm M.A., Xia B., Wu J., Chan F.K., Sung J.J. (2012). Genetics of inflammatory bowel disease in Asia: Systematic review and meta-analysis. Inflamm. Bowel Dis..

[73] Nakazawa A., Dotan I., Brimnes J., Allez M., Shao L., Tsushima F., Azuma M., Mayer L. (2004). The expression and function of costimulatory molecules B7H and B7-H1 on colonic epithelial cells. Gastroenterology.

[74] Beswick E.J., Grim C., Singh A., Aguirre J.E., Tafoya M., Qiu S., Rogler G., McKee R., Samedi V., Ma T.Y. (2018). Expression of Programmed Death-Ligand 1 by Human Colonic CD90(+) Stromal Cells Differs Between Ulcerative Colitis and Crohn’s Disease and Determines Their Capacity to Suppress Th1 Cells. Front. Immunol..

[75] Reynoso E.D., Elpek K.G., Francisco L., Bronson R., Bellemare-Pelletier A., Sharpe A.H., Freeman G.J., Turley S.J. (2009). Intestinal tolerance is converted to autoimmune enteritis upon PD-1 ligand blockade. J. Immunol..

[76] Boutros C., Tarhini A., Routier E., Lambotte O., Ladurie F.L., Carbonnel F., Izzeddine H., Marabelle A., Champiat S., Berdelou A. (2016). Safety profiles of anti-CTLA-4 and anti-PD-1 antibodies alone and in combination. Nat. Rev. Clin. Oncol..

[77] Braga Neto M.B., Ramos G.P., Loftus E.V., Faubion W.A., Raffals L.E. (2020). Use of Immune Checkpoint Inhibitors in Patients With Pre-established Inflammatory Bowel Diseases: Retrospective Case Series. Clin. Gastroenterol. Hepatol. Off. Clin. Pract. J. Am. Gastroenterol. Assoc..

[78] Abu-Sbeih H., Faleck D.M., Ricciuti B., Mendelsohn R.B., Naqash A.R., Cohen J.V., Sellers M.C., Balaji A., Ben-Betzalel G., Hajir I. (2019). Immune Checkpoint Inhibitor Therapy in Patients With Preexisting Inflammatory Bowel Disease. J. Clin. Oncol..

[79] Abu-Sbeih H., Ali F.S., Luo W., Qiao W., Raju G.S., Wang Y. (2018). Importance of endoscopic and histological evaluation in the management of immune checkpoint inhibitor-induced colitis. J. Immunother. Cancer.

[80] Hurwitz H., Fehrenbacher L., Novotny W., Cartwright T., Hainsworth J., Heim W., Berlin J., Baron A., Griffing S., Holmgren E. (2004). Bevacizumab plus irinotecan, fluorouracil, and leucovorin for metastatic colorectal cancer. N. Engl. J. Med..

[81] Sugrue M., Kozloff M., Hainsworth J., Badarinath S., Cohn A., Flynn P., Steis R., Dong W., Sarkar S., Grothey A. (2006). Risk factors for gastrointestinal perforations in patients with metastatic colorectal cancer receiving bevacizumab plus chemotherapy. J. Clin. Oncol..

[82] Wu Y.S., Shui L., Shen D., Chen X. (2017). Bevacizumab combined with chemotherapy for ovarian cancer: An updated systematic review and meta-analysis of randomized controlled trials. Oncotarget.

[83] Burger R.A., Brady M.F., Bookman M.A., Monk B.J., Walker J.L., Homesley H.D., Fowler J., Greer B.E., Boente M., Fleming G.F. (2014). Risk factors for GI adverse events in a phase III randomized trial of bevacizumab in first-line therapy of advanced ovarian cancer: A Gynecologic Oncology Group Study. J. Clin. Oncol. Off. J. Am. Soc. Clin. Oncol..

[84] Fang P., Hu J.H., Cheng Z.G., Liu Z.F., Wang J.L., Jiao S.C. (2012). Efficacy and safety of bevacizumab for the treatment of advanced hepatocellular carcinoma: A systematic review of phase II trials. PLoS ONE.

[85] Read S., Malmström V., Powrie F. (2000). Cytotoxic T lymphocyte-associated antigen 4 plays an essential role in the function of CD25(+)CD4(+) regulatory cells that control intestinal inflammation. J. Exp. Med..

[86] Karandikar N.J., Vanderlugt C.L., Walunas T.L., Miller S.D., Bluestone J.A. (1996). CTLA-4: A negative regulator of autoimmune disease. J. Exp. Med..

[87] Francisco L.M., Sage P.T., Sharpe A.H. (2010). The PD-1 pathway in tolerance and autoimmunity. Immunol. Rev..

[88] Qin S., Xu L., Yi M., Yu S., Wu K., Luo S. (2019). Novel immune checkpoint targets: Moving beyond PD-1 and CTLA-4. Mol. Cancer.

[89] Vey N., Dumas P.Y., Recher C., Gastaud L., Lioure B., Bulabois C.E., Pautas C., Marolleau J.P., Leprêtre S., Raffoux E. (2017). Randomized phase 2 trial of lirilumab (anti-KIR monoclonal antibody, mab) as maintenance treatment in elderly patients (pts) with acute myeloid leukemia (AML): Results of the EFFIKIR trial. Blood.

